# Hospital and Institutional News

**Published:** 1919-04-05

**Authors:** 


### HOSPITAL AND INSTITUTIONAL NEWS.

### THE HEALTH MINISTRY.

SIR ROBERT MORANT, K.C.B., has been designated as First Secretary to the new Health Ministry, and the news should be received with general satisfaction by the profession. At a time when there is apparently so much unrest and lack of unity among the doctors of the country it should inspire the fullest confidence to recall that Sir Robert accepted the chairmanship of the National Insurance Commission in a year when the whole profession was in revolt and when it looked as though the scheme itself was going to prove quite unworkable. He has continued successfully to deal with the situation and develop the maximum available good out of a singularly inelastic panel system until the whole, though still claiming many critics, has grown to an efficiency at one time apparently impossible. Previously for many years Sir Robert Morant did work of the greatest value at the Board of Education, and in making this appointment we think that Dr. Addison has secured a man who has shown a real knowledge of the health problem as a whole, and capable of infusing his own invaluable spirit of progress and efficiency into the work he will undertake.

### COLONEL BARLING'S HOSPITAL SCHEME.

Is it because the once-talked-of scheme for a kind of "King Edward's Hospital" Fund for the Midlands has never been realised that Colonel Gilbert Barling, Vice-Chancellor of Birmingham University, has advocated in the *Birmingham Post* a plan for the migration of the city hospitals to rural districts by means of State or municipal aid? Beginning with the confession that accommodation for in-patients in Birmingham voluntary hospitals had virtually stood still for years, notwithstanding the growth of the population, Colonel Barling argues that "the voluntary system is not able to provide the additional accommodation required." The hospitals, he says, in effect, have their debts only to depend upon, and that indebtedness is likely to increase. Thus, he continues, waiting-lists increase, discharges are premature, and many patients fail to get institutional treatment in time. Every mental and nervous case is virtually unprovided for, and the same want of provision applies to all special departments. Colonel Barling's solution of the problem is the establishment of one great but inexpensively built hospital at some distance from the centre of the city which would include special departments of all kinds. When that proves insufficient, a further migration countrywards could take place. The site he suggests is Bristol Road, just beyond Selly Oak, to which trams run, but which is not built over. The General and the Queen's Hospital would be retained as receiving centres for accidents and cases of urgency. For the sake of medical education, the new hospital system should be affiliated closely with the University. Part of this plan would require the reorganisation of "dental education" by close association between the University and the dental hospitals, so that men and women of intelligence and moderate means may be encouraged to adopt the profession of dental surgery in numbers sufficient to meet the need for the extension of this treatment disclosed by the war.

### MAY 2 IN BIRMINGHAM.

IN connection with Colonel Barling's scheme for the migration of Birmingham hospitals, which we note above, the newly found Hospitals Council has decided to hold a meeting on May 2. On that date the position of the hospitals will be publicly discussed, and the gathering will be addressed by various hospital authorities. We shall thus see if there is any life in the old plan for a hospitals' fund for the Midlands, or whether all this is to be waved aside for the benefit of the proposed Minister of Health.

### THE CLOSING OF MIDDLESEX HOSPITAL.

FROM July 1 till October 1 Middlesex Hospital will be closed. This is not for "spring cleaning," though the war has pushed such matters sadly into the background, but that the engineering plant may be completely overhauled. The cost of this work and the repairs which it may entail is expected to amount to £15,000. While the hospital is shut, cancer patients will continue to be treated at the branch hospital at Clacton.

### AUTOGRAPH LETTERS OF MISS NIGHTINGALE.

ON Friday, April 11, at Messrs. Sotheby, Wilkinson, and Hodge's auction rooms in New Bond Street, a collection of autograph letters by Miss Nightingale is catalogued for sale in seven separate lots. The letters deal largely with nursing matters in Crimean times: one of them explains that, although drunkenness seems inevitable among the nurses of civilian institutions, it should on no account be tolerated amongst those of military hospitals. In a word, the letters are of far greater interest to the nursing profession and the institutional world than they can possibly be to any one else, it may be presumed. If this is the case, it would appear that a great opportunity is open for some liberal-minded man or woman to purchase the whole collection, or at least the most important of the lots, and to present them to some institution or association which will value them and preserve them carefully. The College of Nursing is such a body, though it may not be very feasible as yet to provide a suitable showcase and houseroom for their exhibition. St. Thomas's Hospital would, of course, be an equally appropriate recipient, as, indeed, would also any of the leading voluntary hospitals in the Empire. Given the donor, there should be no difficulty in finding a willing recipient.

### THE PUBLIC SCHOOL HOSPITAL.

ALL Public School boys remember an institution called the "San" to which disease or fear of other consequences would from time to time conduct them. In the epidemics proper to the Lent terms, one or two boys occasionally died there; but the "San" was a place of refuge and cherished accordingly. Of course, the "Sanatorium" was never able to cope with a school epidemic of serious size, but it has needed the influenza to bring this fact home to headmasters. In fact, one of the by-products of that microbic invasion has been the decision of Harrow School to spend £20,000 on a new sanatorium. The School suffered severely with influenza and at one time 300 boys were simultaneously ill.

### GENEROUS OFFER TO BOLTON.

Bolton Infirmary has been offered by the Bleachers' Association as a gift the residence known as Watermillock. The house, which stands on the outskirts of the town, has been used as a hospital for wounded soldiers, and the idea is that the Infirmary should take it over and apply it to the uses of the institution. The proposed gift, it is stated, represents a sum of £40,000. The interesting announcement was made at the annual meeting of the infirmary by Lord Leverhulme who, as president, has himself been a large benefactor of the institution as well as of the town of Bolton, which is his birthplace, and the place in which he laid the foundations of his huge enterprises.

### AN ANTICIPATED LEGACY.

A friend of Wolverhampton and Staffordshire General Hospital, who had formerly promised to leave £6,000 in his will to provide a new out-patients' department, now wishes the building to be accomplished in his life-time. He therefore has said that he will provide the money now, instead of bequeathing it, if the Committee is ready to start on the work.

### THE SEAMEN'S HOSPITAL.

Captain R. E. V. Bax has been appointed Assistant Secretary of the Seamen's Hospital at Greenwich. Prior to the war Captain Bax practised at the Chancery Bar. He joined up in the year 1914 and obtained a commission in the Middlesex Regiment, proceeding to Gallipoli. Captain Bax succeeds Mr. A. P. Hughes Gibb, O.B.E., who is now Secretary to Major Waldorf Astor, M.P.

### THE WILLIAM HEY CENTENARY.

It is just a hundred years ago since William Hey died, the founder of Leeds General Infirmary. A marble statue of him by Chantrey is to be seen in the entrance hall of the Infirmary, and no one who passes before it can doubt how foolish are they who think that memorials to the dead should be confined to bricks and mortar. Hospitals, like other organisms, live mostly by memory, by tradition as we say, and that tradition is fostered by works of art, when an artist is allowed to have a hand in fashioning them. On his mother's side physic was the family's profession. His father was a drysalter of Pudsey. Hey himself became a surgeon, and after studying at St. George's Hospital, began to practise in Leeds at the age of twenty-three, in 1759. Ten years later the General Infirmary was founded, to be finished by Howard, the philanthropist, in 1778, as one of the best hospitals in the country. Hey died in 1819, after forty-four years as its senior surgeon, having retired in 1812.

### A NEW COUNTY COUNCIL SANATORIUM.

A handsome reversion has been announced in Gloucestershire, where the Red Cross Society has promised to give a big private house near the city to the County Council to enable it to carry out its scheme for a large tuberculosis sanatorium. Other plans having happily fallen through, the Society offers the house for twenty-one years, to pay its rent for that period, and to give to the Council all the equipment now at Standish, with more from other hospitals. The value of the latter promise is said to be £3,000. The house having been inspected and approved by the Medical Officer of Health for the county, it remains only to fulfil the condition attached to the gift. This condition is that the Council at first should give priority of admission to ex-soldiers suffering from tuberculosis, a work for which the War Office will pay. Civil patients will be admitted after this obligation has been met.

### THE EAST SUFFOLK MEMORIAL.

To satisfy the desires of county boroughs and of rural districts is the task of most provincial War Memorial Schemes. Ipswich hopes to do so by building a memorial arch in the borough and extending the East Suffolk Hospital. The hospital did a great work during the war at a risk peculiar to its geographical position, which lay in the direct route of invading aeroplanes. If the £80,000 asked for can be raised quickly, the East Suffolk and Ipswich Hospital will take the rank which its work has long justified so far as its difficulties permitted. Its reward will come in having forestalled the aid of a Minister of Health and therefore escaped the conditions which that help will necessarily impose.

### THE FRYS AND SALTAIRE HOSPITAL.

At length, after a patriotic delay on his part, Mr. E. Clifford Fry is carrying out his former intention of resigning the honorary secretaryship of Saltaire Hospital, Yorkshire. For longer than we care to remember, his father, Mr. William Fry, was clerk to the governors; and when the latter died, Mr. Clifford Fry, who had acted as deputy-clerk during his father's last illness, consented to succeed to the post on conditions. The clerkship he declined, but he agreed to act as honorary secretary if a new clerk was appointed. Hence the appointment of Mr. Thomas Luxton. Mr. Fry himself was about to retire when the war began, but because of the difficulties connected with the hospital's use as an auxiliary hospital, he still continued to serve. Now that the institution has reverted to its original work, he feels free, and the thanks of the governors go with him into his retirement. The official diet of the military patients was spiced by extras at Mr. Fry's instance, but this and other memories of his latest work must not obscure his long record of service to civilian patients.

### THE BABIES' HOSPITAL, MANCHESTER.

WE always follow with interest the progress of Manchester Babies' Hospital, which has done enough in five years to show that it has the future of the mustard seed before it. The original experiment began with twelve beds, the third hospital of its special type in the country. Now its thirty beds already overcrowd the house at Levenshulme, and it is now hoped to build a proper hospital with seventy beds and to be content with private houses no more. It began by receiving cases of malnutrition. Its medical staff now wants to receive early cases of rickets. This would mean the admission of infants up to the age of two years, and the allotment of twenty beds out of the seventy to the accommodation of rickety children. The municipal clinics already make use of eighteen beds. Women are trained in infant-care, and some modest research is done into the nutritional diseases of infancy. The building fund is open. It is for Manchester to fill the empty sack.

### TORBAY AND THE TOWER OF BABEL.

WHEN we last visited the Torbay Hospital it was a place which seemed remote from the possibilities of war, and its list of normal patients included no Brazilians, Chinamen, or even negroes. But this exclusiveness has become, at least temporarily, a thing of the past. The Grand Fleet, the men of the M.L. boats, have supplied unexpected patients, and the patients have often been men half-drowned and perhaps wounded by bullets after rescue from torpedoed vessels. The latter have not been confined to the people of Torquay, or even to citizens of the British Empire. They include Brazilians, Danes, Italians, Norwegians, Spaniards, and negroes, after whom the presence of Chinamen seemed a very humdrum affair. The Englishman has the reputation of being a bad linguist, but that this is at least as much due to the rareness with which he hears any other language but his own as to the laziness of the "natives" is wonderfully illustrated at Torbay. The hospital might have become, it would seem, a Tower of Babel. But no; it is a fact that there was always some one who was able to converse. The chairman, Captain Phillpotts, was observed more than once to be talking with a Chinaman in the language of the latter. One is not in the Navy for nothing. But Mr. E. J. V. Husey, a well-tried friend and exemplary secretary, must have added many memories to his association with the hospital of late. Why should not small hospitals have their histories written?

### PROBATIONERS' SALARIES AND THE BED LIMIT.

Two problems confront the Kidderminster Infirmary, and since both, though quite distinct, concern its status and prestige, they may be considered together. Friction has been complained of, exaggeration has had its chance, and, perhaps, a section of the workpeople or its representatives is somewhat restless in mind. The president, Mr. P. Adam, who has been re-elected to the post, is known to believe in giving matters a chance to settle themselves, and deprecates suggestions for drastic reform and the sweeping solutions of facile critics. The responsibility attached to his post commands a respectful consideration for his policy, and we hope the matter is one which tact and time can remedy, for some harm to the hospital has admittedly been done. The second problem is less exciting, but perhaps of deeper importance. It was tersely put by Dr. Evans when he lately stated that a movement was on foot to confine the teaching of probationers to hospitals with at least 100 beds. Of course, the conditions attaching to the granting of a certificate of training have always had regard to the experience open to the person taught. In practice a certificate of value to its possessor has come from those institutions with at least 100 beds. That number has proved a rough test of experience. But it is only a rough one. Number is not enough. It is easier to give a certificate to a nurse than to train her; and the easier way has found its disciples. The movement referred to by Dr. Evans is no doubt an attempt to raise the standard. His point, however, was not concerned with that aspect of the matter. He only wished to suggest that if hospitals with fewer than 100 beds did not add to their number, they might before long be debarred from teaching probationers. The result of such a prohibition would be that the small hospitals would have a higher salaries bill because they could employ no inexpensive probationers, only a staff of fully qualified nurses. The only alternative would be to build. Meantime, the probationer has her peculiar place in the financial scheme of the smaller hospitals. No hospital, however, should seek economy in its salaries' bill, for a voluntary hospital exists to spend money rather than to save it.

### INFLUENZA IN SOUTH AFRICA.

THE Union Government Commission appointed to report on the recent influenza epidemic has issued its report, which was laid before the Union Parliament at Capetown on Tuesday, February 18. The Commission urges that permanent hospitals be provided in the rural areas of the Union, and that a campaign of education be started to remove the prejudice of the rural population against admission to hospital. It also recommends the establishment of hospitals at central points in native areas. During the recent epidemic of influenza in South Africa there were 139,472 deaths, comprising 11,727 Europeans and 127,745 coloured people. The death-rate was highest in the Cape Province, particularly in the Capetown district, which was the first attacked.

### TUBERCULOUS SOLDIERS AS IMMIGRANTS.

IN South Africa it is said that every third bank clerk and every fourth boarding-house inmate is a consumptive. At any rate, the old-fashioned idea of climate for consumption did so abound in the past, and does so still in the lay mind, that the country named has had to pay a penalty of its popularity. Its population has a dangerously large tuberculous contingent, especially when it is remembered that the "Cape boy" and other coloured persons are highly subject to the disease. Very naturally protective legislation, just as in the case of Australia and New Zealand, has been enacted, and it is certain that there will be no wholesale waiving of the regulations in favour of tuberculous soldiers, who, indeed, since many parts of South Africa are practically Dutch speaking, might not readily find their bearings in a strange land. There is a strong feeling in the country, so it is reported, to the above effect.

### THE FUTURE OF MELKSHAM HOSPITAL.

The Jubilee Fund in connection with Melksham Cottage Hospital has been so well supported that the scheme for its extension and alteration only awaits the conclusion of peace for the existing plans to be examined, and probably for building to be soon begun. Even last year there were 113 in-patients; the private wards were well used; and twenty-one cases received free treatment under the Workmen's Scheme. Twenty-seven cases were attended by the midwife: 653 eggs arrived on Egg Sunday, and the only regretted fact of the past year has been the resignation of Sister Way, in whose hands the hospital's reputation has long flourished. Her successor is Sister Bevan, who has now arrived at a time when new developments are pending and consequently with interesting opportunities for her institution and her own career.

### THE PASSING OF THE HOUSE STEWARD

A fraction of an old tradition perishes with the decision of the Committee of Stockport Infirmary to abolish the "house stewards," and in adding to their number to term them the house committee. The weekly meetings of the old ward therefore cease. The house committee will meet "as required," but the house stewards, like hospital beer, are now merely a cherished memory. The new Chairman, under this change, is Sir Alan Sykes, Bart., who has already done good work as honorary treasurer to the hospital. Mr. Pearce, the Superintendent, probably does not regret the change which has brought the hospital into line with other institutions boasting house committees. But "house steward" is an evocative term. Those whose hobby is hospital history receive from it associations, simple or sinister, of hospital life a hundred years ago.

### A SHROPSHIRE WAR MEMORIAL.

It is good news that the Salop Royal Infirmary and the Shropshire Surgical Home have agreed mutually to support the county scheme for a War Memorial with the important corollary that none of the money raised for this purpose shall be spent on the buildings of either existing institution. By this statesmanlike plan, neither will become the other's rival and both will benefit from the proposed new auxiliary hospital which will be in close relation to both. The advantages of co-operation and the benefits to be derived from mutual self-sacrifice could hardly be better illustrated. It is felt that the present buildings of the Salop Royal Infirmary should not be tinkered with, and that the memorial should be a self-contained addition to the county to which every Shropshire lad can point with pride. There is also a proposal for a visible monument, which (it depends on the site) might be placed at the entrance of the proposed auxiliary hospital, or, it is suggested, on top of the Wrekin. The Wrekin is so lovely and distinct as it is that to crown it with a column might easily spoil its symmetry and reduce it to the level of a mound.

### THE FIRE AND ITS LESSON AT LEEDS.

A small hospital fire is always a great matter, and the fact that the one which broke out in the store and dispensary of Leeds General Infirmary last week was actually put out in a quarter of an hour is, in the light of that event, the most interesting part about it. Hospital fires should not occur. Because they do, the following questions must be asked on each occasion: How did it begin; a fact not yet explained. The cotton wool in the store was, of course, highly inflammable; therefore, what special precautions had been taken to protect it? The patients were not allowed to be alarmed. Mr. Leyston, the chairman, and Mr. F. Bray, the general superintendent, were on the spot before the brigade's work was finished. A great escape must be the first verdict on the affair. But that verdict requires the rider that the habitual precautions against fire should be made public; any hitch, however small, on the present occasion should be frankly admitted; and the extent to which (a) fire drills are thoroughly practised, and (b) where, if at all, their sufficiency was wanting should be made known. It is part of the voluntary hospital's standard to regard an avoidable accident as a serious offence; and a fire in hospital should always be avoidable. Only by having that object sternly in view will fires actually be avoided.

### A CAPITAL QUESTION.

The Ashford Cottage Hospital is in need of an X-ray installation. It has been much discussed, but the committee apparently thinks that the money can be found only if it is taken from capital. The capital, however, is in the hands of the Charity Commissioners. An appeal to them is therefore to be made. It is an old situation. Have not our readers an opinion what the decision will be?

### THIS WEEK'S DRUG MARKET.

The downward movement in the prices of drugs continues unabated, and as it continues it involves a still larger number of drugs. This week the following have been marked down in price: caffeine, cocaine, antifebrin, aspirin, barbitone, gallic acid, salicylic acid, sodium salicylate, liquorice juice, amidopyrin, benzaldehyde, creosote carbonate, guaiacol carbonate, lycopodium, clove oil, phenacetin, sodium benzoate, aloin atropine sulphate, chloral hydrate, homatropine, paraldehyde, podophyllin, and thymol. A distinctly easier tendency is noticeable in the prices of various other drugs, and there has been a slight reduction in the quotations for salts of morphine. There does not seem to be any reason to believe that this downward movement is approaching its end, and buyers should continue to act with caution.

